# Identification of the Separation Range of an Incomplete Interlobar Fissure in Segmentectomy Using Near Infrared

**DOI:** 10.7759/cureus.38009

**Published:** 2023-04-23

**Authors:** Yusuke Saeki, Kojiro Nakaoka, Masaharu Inagaki

**Affiliations:** 1 Department of Thoracic Surgery, University of Tsukuba, Tsukuba, JPN; 2 Department of Thoracic Surgery, Tsuchiura Kyodo General Hospital, Tsuchiura, JPN

**Keywords:** near-infrared fluorescence imaging, indocyanine green (icg), incomplete interlobar fissure, segmentectomy, lung cancer

## Abstract

In segmentectomy for patients with incomplete interlobar fissures, insufficient dissection of the interlobar parenchyma may result in incomplete segmentectomy, while excessive dissection may lead to excessive bleeding and air leaks. Here, we report a case of left apicoposterior (S^1+2^) segmentectomy with incomplete interlobar fissure in which near-infrared thoracoscopy with indocyanine green was used to identify the separation range of interlobar fissure by dissecting the relevant vessels beforehand.

## Introduction

In anatomical lung resection in patients with incomplete interlobar fissure, dissection of the interlobar parenchyma can cause hemorrhage and air leaks. Fissureless techniques, which involve dissecting the lung parenchyma using mechanical staplers at the final step after dividing hilar bronchovascular structures, are used in lobectomy to avoid dissecting the interlobar parenchyma [1]. However, this technique is difficult to apply to segmentectomy because partial interlobar dissection is required and has been rarely reported. Insufficient dissection may result in incomplete segmentectomy, while excessive dissection can lead to bleeding and air leaks.

Near-infrared (NIR) thoracoscopy with indocyanine green (ICG) is used for intersegmental plane identification in segmentectomy [2]. This technique involves intravenously injecting ICG after the vascular dissection of the segment to be resected and then observing the fluorescence with NIR thoracoscopy. This enables the visualization of the intersegmental plane, as the segments are preserved fluoresces, while the resected segment remains unenhanced. In addition to intersegmental plane identification, NIR thoracoscopy has also been reported to be used for the identification of the interlobar fissure boundary in lobectomy for a patient with severe incomplete interlobar fissure [3].

We report a case of left apicoposterior (S^1+2^) segmentectomy using NIR thoracoscopy with ICG to identify the separation range of interlobar fissure after dissecting the relevant vessels.

## Case presentation

**Figure 1:**
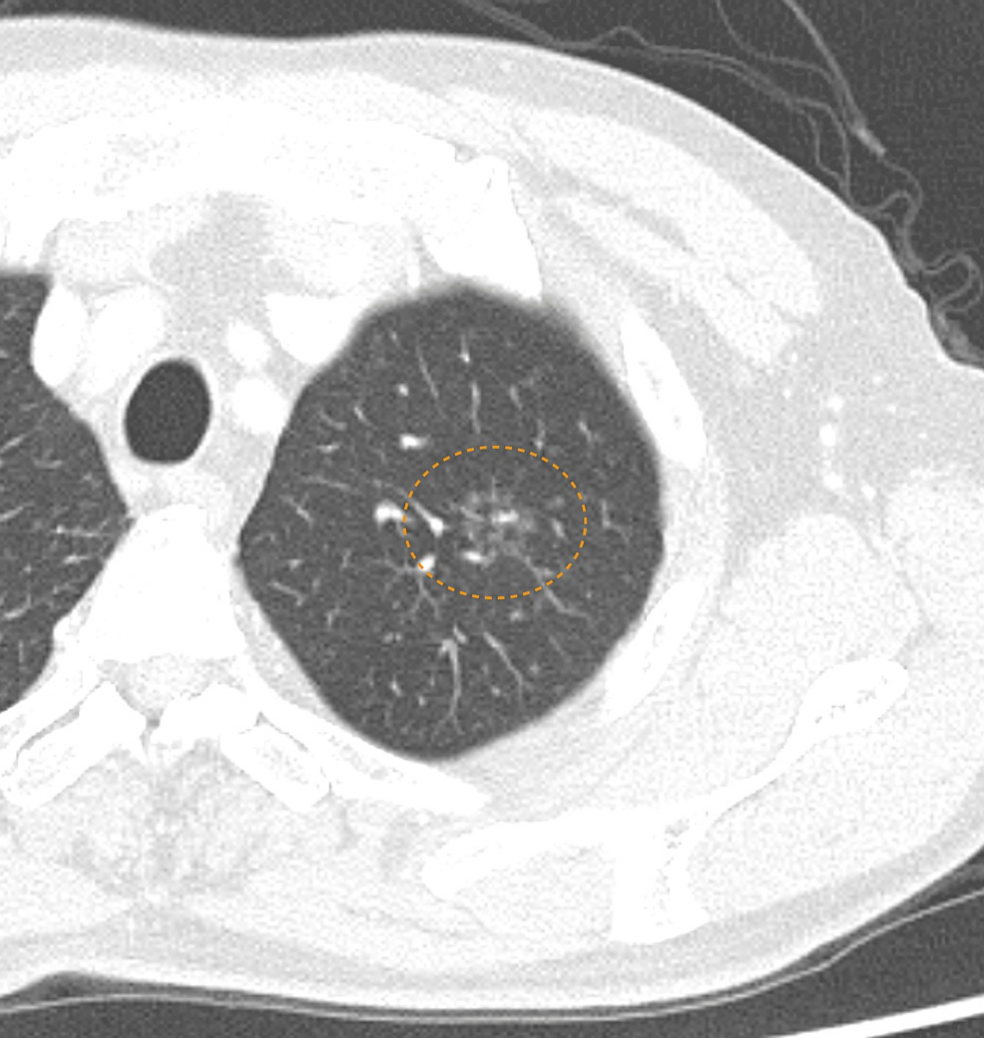
Preoperative chest CT Preoperative chest CT reveals a partially solid, ground-glass nodule containing a solid component in the left apicoposterior segments (S^1+2^).

The patient was a 74-year-old man with stage IA1 lung cancer. CT showed a partially solid, ground-glass nodule measuring 15 mm (with the solid component measuring 3 mm) in the left apicoposterior segments (S^1+2^) (Figure 1).

**Figure 2:**
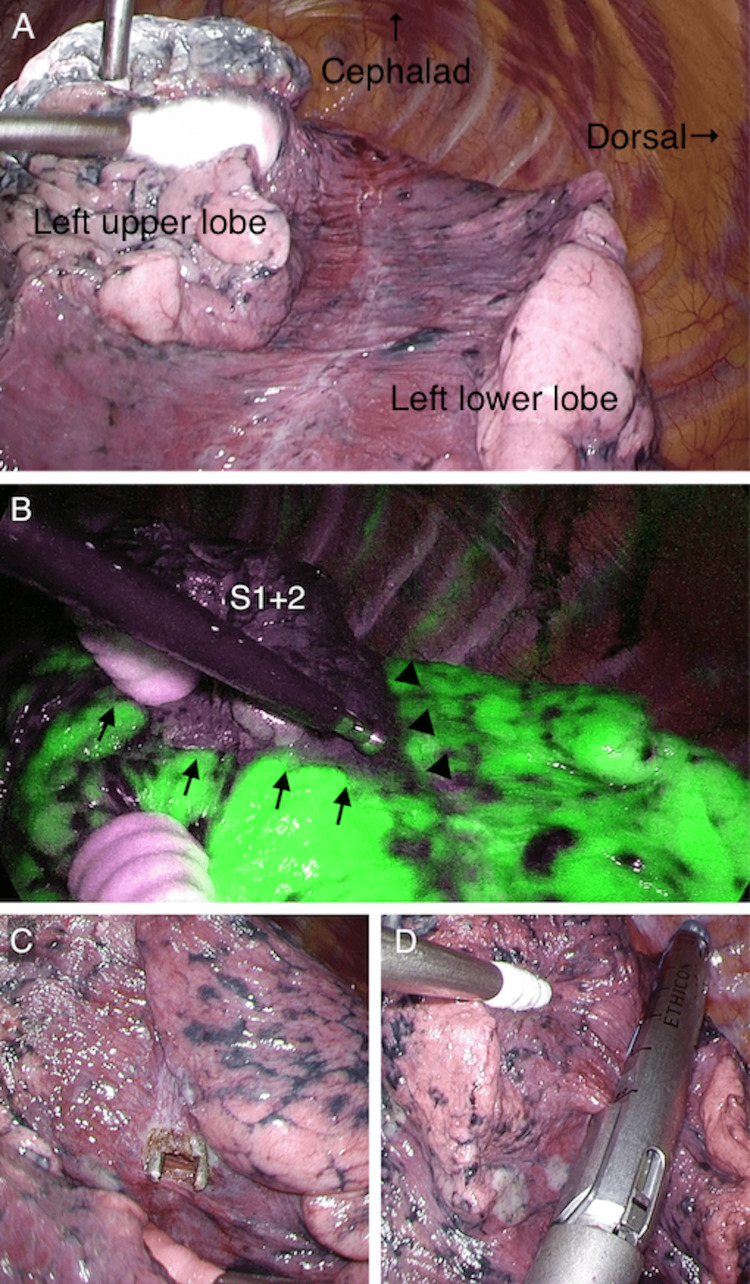
Intraoperative images A. This image shows that the interlobar fissure is incomplete. B. NIR thoracoscopy with ICG reveals the intersegmental plane (arrows) and the separation range of the interlobar fissure (arrowheads). C. Interlobar tunneling is performed using forceps from the dorsal pulmonary hilum. D. The interlobar fissure is separated using mechanical staplers.

Video-assisted thoracoscopic surgery left S^1+2^ segmentectomy was performed via a 4-cm access incision at the fourth intercostal space using a two-port incision. The pulmonary artery was not visible due to an incomplete interlobar fissure (Figure 2a). The apicoposterior venous branches (V^1+2^) and the apicoposterior artery branches (A^1+2a+b^ and A^1+2c^) were dissected clockwise from the ventral side to the dorsal side of the pulmonary hilum. After intravenous ICG (0.3mg/kg/10ml) bolus administration, NIR thoracoscopy (VISERA ELITE II, Olympus, Tokyo, Japan) identified the intersegmental plane and the separation range of interlobar fissure (Figure 2b). The limits of the segments and the interlobar fissure were marked by electrocautery. Interlobar tunneling was performed with the forceps from the dorsal pulmonary hilum using the markings as a guide (Figure 2c), and the interlobar fissure was separated using mechanical staplers (Figure 2d). Following this, the apicoposterior bronchus (B^1+2^) was dissected, and finally, the intersegmental plane was created using mechanical staplers, completing the left S^1+2^ segmentectomy. No intraoperative air leaks occurred. The patient experienced no complications and was discharged 10 days after surgery.

## Discussion

NIR thoracoscopy with ICG in thoracic surgery is a relatively new technique, initially used for intersegmental plane identification in segmentectomy [2] and recently for the identification of the interlobar fissure boundary in lobectomy for a patient with severe incomplete interlobar fissure [3]. However, there are few reports on the identification of the separation range of interlobar fissures in segmentectomy with incomplete interlobar fissures using NIR thoracoscopy with ICG [4]. We believe that the method we employed in this study is useful and warrants reporting.

This method has the advantage of minimizing bleeding and air leaks while still allowing for essential and sufficient interlobar dissection. It has been reported that the frequency of pulmonary fistulas and the local relapse rate in segmentectomy are higher than in lobectomy [5]. This approach enables the precise identification of the separation range of interlobar fissures, reducing pulmonary fistula incidence and the risk of insufficient margins.

The key aspect of this method is the preliminary dissection of relevant pulmonary arteriovenous vessels. Administering ICG after dissecting the relevant vessels allows identification of the intersegmental plane and the separation range of interlobar fissure. On the other hand, this method is unsuitable for cases where dissection of the relevant vessels is difficult to perform. NIR thoracoscopy with a transbronchial injection of ICG has been reported [6] and may be effective in such cases.

Finally, we would like to mention two points of caution regarding the use of NIR thoracoscopy with ICG intravenous injection. The first point is that administration is contraindicated in cases with a known allergy to the contrast agent. Moreover, while ICG is generally regarded as safe, serious adverse reactions have been recorded in 0.05% of cases [7]. The second point is that the staining time with ICG is relatively short, approximately 90 seconds after intravenous injection [8], and there is a probability of fluorescence spreading outside the resection segment [9]. Therefore, it is essential to carefully perform intersegmental marking at the appropriate timing to avoid incomplete segmental resection.

## Conclusions

In S^1+2^ segmentectomy with incomplete interlobar fissure, by dissecting relevant vessels first, the separation range of interlobar fissure was easily identified using NIR thoracoscopy with ICG. This technique is considered to be useful in reducing the risk of hemorrhage and air leaks.
